# Identification of perception gaps between physicians and patients with neurological diseases and the prediction of these gaps using machine learning

**DOI:** 10.1038/s41598-025-33500-x

**Published:** 2026-02-09

**Authors:** Genko Oyama, Yuji Tomizawa, Taiji Tsunemi, Shuko Nojiri, Taku Hatano, Wataru Sako, Yasunobu Hoshino, Shin-ichi Ueno, Daiki Kamiyama, Yutaka Oji, Ayami Okuzumi, Daisuke Taniguchi, Haruna Haginiwa, Takuma Maeda, Yoshihiko Furusawa, Miwa Izutsu, Nobutaka Hattori

**Affiliations:** 1 Department of Neurology, Faculty of Medicine, Juntendo University, 2-1-1 Hongo, Bunkyo-ku, Tokyo 113-8421 Japan; 2 Medical Technology Innovation Center, Juntendo University Faculty of Medicine, Bunkyo-ku, Tokyo Japan; 3 Hash Peak K.K., Chuo-ku, Tokyo Japan; 4 Takeda Pharmaceuticals Company Limited, Chuo-ku, Tokyo Japan

**Keywords:** Machine learning, Neurological diseases, Patient-centered care, Perception gaps, Shared decision-making, Neurology, Epilepsy, Multiple sclerosis, Parkinson's disease

## Abstract

Understanding perception and communication gaps between patients with neurological diseases and their treating physicians is essential for optimizing patient-centered care. The GAP-AI study aimed to identify these gaps in a cohort of patients with Parkinson’s disease, multiple sclerosis, or epilepsy. This single-center observational study involved patients (N = 197) and their treating physicians (N = 12) answering questionnaires (18-item Patient Satisfaction Questionnaire Short Form, 9-item Shared Decision Making Questionnaire for patients and physicians, Barthel Index, and 36-item Short Form subdomains) over two clinic visits. The primary outcome was the difference between pairwise items in the questionnaires (perception gap). Perception gaps, albeit minimal, were identified for patient satisfaction, shared decision-making, activities of daily living, and quality of life. Attributes that significantly influenced perception gaps included physician’s age, years of experience/holding a neurologist qualification, disease area, and the number of patients treated, with experienced physicians tending to provide more rigorous evaluations than their patients’ self-assessments. Multiple machine learning algorithms were used to develop predictive models based on study data. The k-nearest neighbors algorithm demonstrated the best performance in predicting a patient–physician perception gap. Insights from our study highlight the potential to recognize, predict, and ultimately address these gaps, thus enhancing clinical practice by increasing the level of understanding between patients and their physicians.

**Supplementary Information:**

The online version contains supplementary material available at 10.1038/s41598-025-33500-x.

## Introduction

Neurological diseases, such as Parkinson’s disease, multiple sclerosis, and epilepsy are chronic in nature with complex symptomology. As these diseases progress, their negative impacts on activities of daily living (ADLs), and thus quality of life (QoL), become increasingly severe^1–4^.

Understanding the complex symptoms of neurological diseases, which constantly evolve, and how they affect daily functioning is important for effective decision-making in clinical practice, which in turn influences patient treatment satisfaction. Recently, ‘shared decision-making’ (SDM), whereby healthcare professionals and patients share evidence and determine treatment decisions together, has been recognized as playing an important role in achieving patient treatment satisfaction^5^. SDM encompasses patient-centered care by incorporating both the patient’s pathologic condition and clinical symptoms (from the viewpoint of a physician) and the patient’s preferences, values, and goals. Implementation of SDM has been reported to improve patient outcomes and reduce medical expenses^6,7^. However, most clinical decisions are based on information obtained only at the time of medical examination, thus limiting physicians’ ability to grasp the full spectrum of patients’ symptoms and their impacts on daily life. It therefore seems likely that perception gaps exist between patients and physicians in terms of their communication, and recognition of disease status and treatment goals. Studies have identified such gaps in both non-neurological^8–10^ and neurological disease settings^10–15^.

Perception gaps have been identified between patients with Parkinson’s disease and their physicians in terms of the relative importance of different disease symptoms and treatment delivery options, and their knowledge of support networks^11,12^. In addition, some aspects of QoL have been rated as more important by patients with multiple sclerosis than by their treating physicians^14^. Similarly, patients with epilepsy in another study rated reducing seizure severity as a more important treatment goal than seizure frequency, which was reversed with physicians^15^. Patients with multiple sclerosis have also self-reported more relapses than physician-documented relapses during routine clinical practice, and perception gaps have been found to be more pronounced in patients with greater disability or decreased treatment satisfaction^13^ and in certain ethnicities^10^.

Most studies have compared perception gaps in disease recognition and communication indirectly between groups of patients and physicians. However, studies that have directly identified perception gaps between patients with neurological diseases and their treating physicians are limited, particularly in Japan^12,15^. An evidence-based, patient-centric approach that takes into consideration the characteristics of individual patients and their physicians is required to optimize their outcomes^16^. Hence, understanding the gaps in the overall management of neurological diseases is an area warranting further research. We hypothesized that patient and physician factors can influence whether a perception gap exists.

There is increasing evidence that artificial intelligence (AI) and machine learning could be used to enhance the management of neurological diseases^17,18^. For example, machine learning algorithms trained on patient survey responses can help identify patients at risk of responding negatively to a survey^19^, and models trained on patient self-reported surveys and electronic health records can predict patient satisfaction^20^. We hypothesized that machine learning can generate a predictive model for identifying gaps in perception and communication during patient–physician consultations.

The aim of this observational study was to investigate the perception gaps between patients and physicians in the neurological disease setting and to develop a predictive model using AI (GAP-AI study). The objective of this study was to identify perception gaps between patients with Parkinson’s disease, multiple sclerosis, or epilepsy, and their treating physicians regarding patient satisfaction, SDM, assessment of ADLs, and QoL. Additionally, we investigated factors influencing these gaps and whether they could be predicted using an AI machine learning model.

## Results

### Patient characteristics

This study was conducted between January 2023 and August 2023. In total, 198 patients were enrolled and 197 patients were included for subsequent analyses (one patient withdrew their consent). Of these patients, the mean (SD) age was 58.1 (16.4) years and most were women (60.4%; Table 1). The most common diagnosis was Parkinson’s disease (69.5%), followed by multiple sclerosis (20.3%) and epilepsy (10.2%), with an overall mean disease duration of 7.4 years. Over one-third of patients had their disease for less than 5 years (38.0%). Most patients reported living with someone (82.4%) and not requiring caregiver assistance (61.0%). The mean 36-item Short Form (SF-36) Physical Component Summary (43.2) and Role/Social Component Summary (46.8) scores were slightly below the average of 50 in the general population, whereas the Mental Component Summary (51.4) score was slightly above the average of 50. Most patients had mild-to-moderate disease severity of Parkinson’s disease, multiple sclerosis, or epilepsy (Supplementary Table 1).

**Table 1:** Patient demographics and baseline characteristics.

	N = 197
Age, years, mean (SD)	58.1 (16.4)
Age group, n (%)	
< 30 years	15 (7.6)
30–39 years	12 (6.1)
40–49 years	29 (14.7)
50–59 years	37 (18.8)
60–69 years	50 (25.4)
≥ 70 years	54 (27.4)
Female, n (%)	119 (60.4)
Diagnosis, n (%)	
Parkinson’s disease	137 (69.5)
Multiple sclerosis	40 (20.3)
Epilepsy	20 (10.2)
Disease duration, n (%)^a^	
< 5 years	71 (38.0)
5–10 years	55 (29.4)
≥ 10 years	61 (32.6)
Number of hospital visits in the last 3 months, n (%)^a^	
1	68 (36.4)
2	82 (43.9)
3	21 (11.2)
≥ 4	16 (8.6)
SF-36 component scores, mean (SD)	
Physical Component Summary	43.2 (12.0)
Mental Component Summary	51.4 (9.3)
Role/social Component Summary	46.8 (11.2)
Living with someone, n (%)^a^	154 (82.4)
Requires assistance of caregivers, n (%)^a^	73 (39.0)

^a^n = 187.

SD, standard deviation; SF-36, 36-item Short Form.

### Physician characteristics

Half the physicians were between the ages of 35 and 44 years and most (83.3%) were men (Table 2). More than 40% of physicians had over 20 years of experience and most specialized in Parkinson’s disease (75.0%). Of the 11 physicians (91.7%) who were certified neurologists by the Japanese Society of Neurology, one-third had held their certification for 5–10 years. Most physicians (75.0%) had treated fewer than 500 cumulative patients in their career and one physician had treated more than 2000 patients.

**Table 2:** Physician demographics.

	N = 12
Age group, n (%)	
25–34 years	1 (8.3)
35–44 years	6 (50.0)
45–54 years	5 (41.7)
Male, n (%)	10 (83.3)
Years of experience, n (%)	
5–10 years	2 (16.7)
10–15 years	4 (33.3)
15–20 years	1 (8.3)
≥ 20 years	5 (41.7)
Disease specialist, n (%)	
Parkinson’s disease	9 (75.0)
Multiple sclerosis	2 (16.7)
Epilepsy	1 (8.3)
Cumulative number of patients treated, n (%)	
< 500 patients	9 (75.0)
< 1000 patients	1 (8.3)
< 1500 patients	1 (8.3)
< 2000 patients	0
≥ 2000 patients	1 (8.3)

### Perception gaps in pairwise questionnaire items

The mean (standard deviation [SD]) sum of relative difference between patient and physician responses (Yr) to the 18-item Patient Satisfaction Questionnaire Short Form (PSQ-18), 9-item Shared Decision Making Questionnaire for patients (SDM-Q-9) and physicians (SDM-Q-Doc), Barthel Index, and original questionnaire were 3.4 (9.8), 7.2 (12.2), −0.3 (9.2), and 4.4 (6.4), respectively (Table 3). The mean (SD) sum of the absolute difference between patient and physician responses (Ya) to the PSQ-18, SDM-Q-9/SDM-Q-Doc, Barthel Index, and original questionnaire were 16.3 (5.7), 12.7 (7.8), 3.6 (9.4), and 8.4 (3.9), respectively.

**Table 3:** Perception gaps between patients and physicians in individual questionnaires.

	PSQ-18 (n = 177)	SDM-Q-9/SDM-Q-Doc (n = 185)	Barthel Index (n = 178)	Original questionnaire (n = 186)	SF-36 subdomains (n = 187)
Sum of the relative difference between patient and physician responses (Yr)^a^					
Mean (SD)	3.4 (9.8)	7.2 (12.2)	–0.3 (9.2)	4.4 (6.4)	–
Median (range)	3.0 (–20, 35)	7.0 (–23, 40)	0 (–50, 50)	4.5 (–12, 19)	–
Sum of the absolute difference of patient and physician responses (Ya)^b^					
Mean (SD)	16.3 (5.7)	12.7 (7.8)	3.6 (9.4)	8.4 (3.9)	–
Median (range)	15 (1, 35)	11 (2, 40)	0 (0, 50)	8 (1, 20)	–
Classification of difference, n (%)					
Concordant	91 (51.4)	102 (55.1)	140 (78.7)	96 (51.6)	36 (19.3)^c^
Discordant	86 (48.6)	83 (44.9)	38 (21.3)	90 (48.4)	151 (80.7)

^a^Yr refers to the sum of the patient scores subtracted from the physician scores for each item. A positive value indicates the patient’s score is greater than the physician’s score and vice versa, which in turn infers directionality.

^b^Ya is the absolute value of the sum of patient scores subtracted from the physician scores for each item and was used to assess the degree of concordance in perception between patients and physicians.

^c^When patients and physicians identified the same SF-36 subdomain as the most important to the patient’s QoL, this was considered a match and they were included in the concordant group.

PSQ-18, Patient Satisfaction Questionnaire-18;

QoL, quality of life; SD, standard deviation; SDM-Q-9, 9-item Shared Decision Making Questionnaire from the patient perspective; SDM-Q-Doc, Shared Decision Making Questionnaire from the physician perspective; SF-36, 36-item Short Form.

Overall, 19.3% of patients’ SF-36 subdomain responses matched those of their physicians (Table 3). The subdomains most frequently prioritized by patients were role physical (25.4%), physical functioning (19.3%), and general health (18.8%). These SF-36 subdomains were also most frequently prioritized by physicians, but at different rates: 39.0%, 12.8%, and 15.5%, respectively.

Comparison of the total scores of patient and physician responses to each of the questionnaires, as well as the degree of correlation between the responses, is another approach to evaluating perception gaps. There were significant differences between the mean total scores of patients (Yp) and physicians (Yi) for PSQ-18, SDM-Q-9/SDM-Q-Doc, and the original questionnaire (*P* < 0.001; Supplementary Table 2). Similarly, there were low correlations between patient and physician responses to the individual items of the PSQ-18 (κ = 0.030), SDM-Q-9/SDM-Q-Doc (κ = 0.021), Barthel Index (κ = 0.172), the original questionnaire (κ = 0.039), and SF-36 subdomains (κ = 0.203).

### Factors that influenced perception gaps

Cross-tabulation analyses identified the following patient attributes that significantly influenced the perception gap between the concordant and discordant groups for PSQ-18: caregiver status (present vs absent), age group, and the type of diagnosis (all *P* ≤ 0.002); for SDM-Q-9/SDM-Q-Doc: age group (*P* = 0.020), type of diagnosis (*P* < 0.001), and frequency of hospital visits (*P* = 0.002); for the Barthel Index: caregiver status (*P* = 0.008), age group (*P* = 0.003), occupation (*P* = 0.001), method of transportation to the hospital (*P* < 0.001), and duration of disease (*P* = 0.001); for the original questionnaire: age group (*P* = 0.025) and type of diagnosis (*P* = 0.017); and for SF-36 subdomains: type of diagnosis (*P* = 0.030; Table 4).

**Table 4:** Cross-tabulation analyses of patient and physician attributes that influenced the perception gap.

	Patient attributes		Physician attributes	
	Variants	Fisher’s exact test	Variants	Fisher’s exact test
PSQ-18	Presence of caregivers Age group Type of diagnosis	0.001 0.002 < 0.001	Age group Years of experience as a physician Disease area Years of holding a neurologist qualification Years of experience in the treatment of the target disease Cumulative number of patients treated	0.005 0.001 < 0.001 < 0.001 0.001 < 0.001
SDM-Q-9/SDM-Q-Doc	Age group Type of diagnosis Frequency of hospital visits	0.020 < 0.001 0.002	Age group Years of experience as a physician Disease area Years of holding a neurologist qualification Years of experience in the treatment of the target disease	< 0.001 0.001 < 0.001 < 0.001 0.040
Barthel Index	Presence of caregivers Age group Occupation Transportation method to the hospital Duration of disease	0.008 0.003 0.001 < 0.001 0.001	Years of holding a neurologist qualification Years of experience in the treatment of the target disease Cumulative number of patients treated	< 0.001 0.015 < 0.001
Original questionnaire	Age group Type of diagnosis	0.025 0.017	Age group Years of experience as a physician Disease area Years of holding a neurologist qualification Years of experience in the treatment of the target disease Cumulative number of patients treated	0.017 0.027 0.017 0.015 0.020 0.039
SF-36 subdomains	Type of diagnosis	0.030	Disease area Years of experience in the treatment of the target disease	0.030 0.044

PSQ-18, Patient Satisfaction Questionnaire-18; SDM-Q-9, 9-item Shared Decision Making Questionnaire from the patient perspective; SDM-Q-Doc, Shared Decision Making Questionnaire from the physician perspective; SF-36, 36-item Short Form.

Physician attributes that significantly influenced the perception gap in the five outcomes were similar across the questionnaires and included: physician’s age, years of experience, disease area, years holding a neurologist qualification, years of experience in treating the target disease, and cumulative patients treated (Table 4).

Multiple regression analyses across the PSQ-18, SDM-Q-9/SDM-Q-Doc, and Barthel Index for all patients identified patient age, years of experience as a physician, years of holding a neurologist qualification, physician-reported time for outpatient consultation, and caregiver status as independent variables that significantly influenced the perception gap (Table 5).

**Table 5:** Multiple regression analysis of the independent variables that influence the perception gap.

	PSQ-18			SDM-Q-9/SDM-Q-Doc			Barthel Index		
	Variable	β value	*P* value	Variable	β value	*P* value	Variable	β value	*P* value
All patients^a^	Years of experience as a physician	0.605	0.003	Original Questionnaire_patient total score	0.188	0.031	SDM_patient total score	−0.187	0.010
				Original Questionnaire physician total score	−0.191	0.011	SF-36 subdomain_patient	−0.163	0.032
				Patient_age	−0.239	0.001	Outpatient consultation hours	0.156	0.048
				Years of experience as a physician	−0.880	0.005	Presence of caregivers	0.189	0.011
				Years of holding a neurologist qualification	0.579	0.003			
Parkinson’s disease^b^	Years of experience as a physician	0.200	0.026	Original Questionnaire_patient total score	0.247	0.003	PDQ-39_summary index	0.392	< 0.001
	Outpatient consultation hours	−0.180	0.044	Years of holding a neurologist qualification	−1.396	0.004	Hoehn and Yahr stage	0.223	0.011
				Years of experience in the treatment of the target disease	0.435	0.031			
				Cumulative number of patients treated	0.707	0.001			
				Outpatient consultation hours	–0.578	0.001			
Multiple sclerosis^c^	–	–	–	–	–	–	EDSS	0.513	0.004
Epilepsy^d^	–	–	–	Patient_annual income	−0.441	0.024	N/A^e^		

^a^Patient numbers: PSQ-18 (n = 177), SDM-Q-9/SDM-Q-Doc (n = 185), Barthel Index (n = 178).

^b^Patient numbers: PSQ-18 (n = 121), SDM-Q-9/SDM-Q-Doc (n = 129), Barthel Index (n = 121).

^c^Patient numbers: PSQ-18 (n = 38), SDM-Q-9/SDM-Q-Doc (n = 38), Barthel Index (n = 38).

^d^Patient numbers: PSQ-18 (n = 18), SDM-Q-9/SDM-Q-Doc (n = 18), Barthel Index (n = 19).

^e^For patients with epilepsy, multiple regression analysis was omitted because all items were not significant based on Spearman’s correlation coefficient.

EDSS, Expanded Disability Status Scale; PDQ-39, Parkinson’s Disease Questionnaire-39; PSQ-18, Patient Satisfaction Questionnaire-18; SDM-Q-9, 9-item Shared Decision Making Questionnaire from the patient perspective; SDM-Q-Doc, Shared Decision Making Questionnaire from the physician perspective; SF-36, 36-item Short Form.

Independent variables that significantly influenced the perception gap in Parkinson’s disease included years of experience as a physician, years of holding a neurologist qualification, years of experience in the treatment of Parkinson’s disease, cumulative number of patients treated, time allocated for outpatient consultations, and patient’s disease stage (Hoehn and Yahr; Table 5). Similarly, patient’s disability status (Expanded Disability Status Scale [EDSS]) was an independent variable that influenced the perception gap in patients with multiple sclerosis, and patient’s annual income for patients with epilepsy. Multiple regression analysis was not performed for the Barthel Index in patients with epilepsy as none of the variables showed significance in the univariate analysis.

### Correlation of perception gaps between questionnaires

There were significant correlations between the mean Ya in PSQ-18 and SDM-Q-9/SDM-Q-Doc (ρ = 0.285; *P* < 0.001); PSQ-18 and the original questionnaire (ρ = 0.295; *P* < 0.001); and SDM-Q-9/SDM-Q-Doc and the original questionnaire (ρ = 0.400; *P* < 0.001; Supplementary Table 3). However, there were no significant correlations between patient and physician SF-36 subdomain rankings and the Ya of the PSQ-18 (*P* = 0.488), SDM-Q-9/SDM-Q-Doc (*P* = 0.758), Barthel Index (*P* = 0.829), or the original questionnaire (*P* = 0.701) (Supplementary Table 4).

### Model performance

Multiple machine learning algorithms were evaluated to ensure robust cross-testing. The log loss values for the holdout set among all tested algorithms are shown in Supplementary Table 5 and the area under the curve (AUC) of the receiver operating characteristics (ROC) curves are shown in Supplementary Fig. S1. The k-nearest neighbors model showed the best performance (i.e., smallest log loss values for the holdout set) in predicting the presence or absence of a perception gap identified in the primary outcome from PSQ-18, SDM-Q-9/SDM-Q-Doc, Barthel Index, the original questionnaire, and the SF-36 subdomain of interest (Table 6).

**Table 6:** Model performance of the k-nearest neighbors**.**

	PSQ-18	SDM-Q-9/SDM-Q-Doc	Barthel Index	Original questionnaire	SF-36 subdomains
Holdout log loss	0.1342	0.1709	0.0581	0.1333	0.0500
MCC	0.8100	0.1600	0.2500	0.4200	0.7600
AUC–ROC	0.9888	0.9846	0.9954	0.9907	1.0000
Accuracy	0.9697	0.9444	0.9697	0.9444	1.0000
Precision	1.0000	0.9000	0.9000	0.8947	1.0000
Sensitivity	0.9310	1.0000	1.0000	1.0000	1.0000
Specificity	1.0000	0.8889	0.9583	0.8947	1.0000

AUC, area under the curve; MCC, Matthew’s correlation coefficient; PSQ-18, Patient Satisfaction Questionnaire-18; ROC, receiver operating characteristic; SDM-Q-9, 9-item Shared Decision Making Questionnaire from the patient perspective; SDM-Q-Doc, Shared Decision Making Questionnaire from the physician perspective; SF-36, 36-item Short Form.

**Fig. 1:**
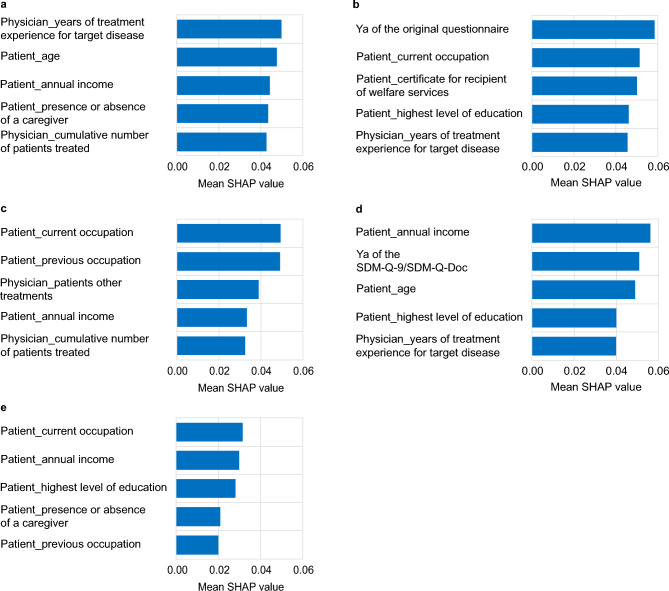
Highest impact features for the specified target measured by SHAP values for the k-nearest neighbors model. (**a**) PSQ-18, (**b**) SDM-Q-9/SDM-Q-Doc, (**c**) Barthel index, (**d**) original questionnaire (**e**) SF-36 subdomains.PSQ-18, Patient Satisfaction Questionnaire-18; SDM-Q-9; 9-item Shared Decision Making Questionnaire from the patient perspective; SDM-Q-Doc, Shared Decision Making Questionnaire from the physician perspective; SF-36, 36-item Short Form; SHAP, SHapley Additive exPlanations; Ya, sum of the absolute difference between patient and physician responses.

The feature importance was measured by SHapley Additive exPlanations (SHAP) values. The top five important features of the k-nearest neighbors predictive model for identifying a perception gap are shown in Fig. 1. For PSQ-18, these features were ‘years of treatment experience for target disease’, ‘patient age’, ‘annual income’, ‘presence or absence of a caregiver’, and ‘cumulative number of patients treated’. For SDM-Q-9/SDM-Q-Doc, the features were ‘Ya for the original questionnaire’, ‘current occupation’, ‘certificate for recipient of welfare services’, ‘highest level of education’, and ‘years of treatment experience for target disease’. For the Barthel Index, the features were ‘current occupation’, ‘previous occupation’, ‘patient’s other treatments’, ‘annual income’, and ‘cumulative number of patients treated’. For the original questionnaire, the features were ‘annual income’, ‘Ya for the SDM-Q-9/SDM-Q-Doc questionnaires’, ‘patient age’, ‘years of treatment experience for target disease’, and ‘highest level of education’. For the SF-36 subdomains, the features were ‘current occupation’, ‘annual income’, ‘highest level of education’, ‘presence or absence of a caregiver’, and ‘previous occupation.’

## Discussion

This is the first study to examine the perception gaps between patients with neurological diseases (Parkinson’s disease, multiple sclerosis, and epilepsy) and their treating physicians using validated patient-reported outcomes (PROs)/observer-reported outcomes (OROs) and an original questionnaire, and to develop a predictive model using machine learning for recognizing these gaps. Perception gaps between patients and physicians have previously been reported in neurological diseases, particularly in the perceived importance of symptoms, relapse reporting, and QoL determinants^11–14^. Such discrepancies can compromise treatment decisions, patient satisfaction, and overall care quality. For example, discrepancies in what patients and physicians prioritize for QoL can skew assessments, while differences in relapse reporting may lead to under- or overtreatment. Addressing these differences is therefore essential for improving communication and fostering patient-centered care. Although studies have shown that perception gaps exist, few have evaluated the reasons behind a perception gap in neurological diseases.

In our study, we identified perception gaps in patient satisfaction, SDM, ADLs, and QoL. These were evident across multiple instruments including the PSQ-18, SDM-Q-9/SDM-Q-Doc, and Barthel Index, with poor agreement between patient and physician responses. Factors that significantly influenced the perception gap in patient satisfaction were caregiver status, disease severity, type of diagnosis, years with a specialist qualification, and consultation time. Similarly, factors that significantly influenced the perception gap in SDM were caregiver status, patient age, type of diagnosis, age of the physician, years of neurologist qualification, cumulative total patients treated, consultation time, and years of experience with the relevant disease. For patient QoL, caregiver status, disease severity, type of diagnosis, and consultation time significantly influenced the perception gap. In a previous cross-sectional survey, differences in relapse reporting in patients with multiple sclerosis and their physicians were observed, and this perception gap was more pronounced in patients with greater disability levels, decreased QoL or treatment satisfaction^13^. The finding that severity affects QoL is consistent with the results of this study.

There is limited research examining the factors that influence perception gaps from a physician perspective. In our study, experience-related factors of physicians were frequently seen as strong predictors in correlation analyses, multiple regression analyses, and machine learning. Physicians with more clinical experience may provide more rigorous evaluations compared with patients’ responses, generating a perception gap. While perception gaps are often viewed negatively, our findings suggest that they may reflect a more cautious or more uncompromising clinical stance by experienced physicians. Another possible reason for this disconnect is that patients may be relatively satisfied with their current management (as these neurological diseases are chronic and progressive) and not seek further improvement in their disease status. This interpretation is important, as the presence of a perception gap does not necessarily indicate poor patient care but may highlight differing priorities or expectations; therefore, it would be meaningful to investigate whether perception gaps affect patient care in the future.

The original questionnaire developed for this study captured aspects of the patient–physician relationship not addressed by standard tools. Hence, some items in this questionnaire could potentially be used as scales to measure perception gaps that are not included in other validated questionnaires, such as communication status and trust between patients and their treating physicians, and clinical environment.

The strong performance of the k-nearest neighbors algorithm in this study can be attributed to its non-parametric nature, meaning it does not rely on assumptions about the underlying data distribution or linear relationships. Instead, it makes predictions based on the proximity of data points, allowing it to adapt to the local structure of the data. This characteristic is particularly beneficial in scenarios with limited data, such as in our study, where it can effectively leverage local patterns and similarities to make accurate inferences. While our machine learning model demonstrated strong predictive performance in identifying perception gaps, we acknowledge the ethical imperative to address potential biases inherent in AI systems. As highlighted in recent literature, such biases can arise from non-representative training data, underrepresentation of minority groups, or unclear model development processes, potentially leading to prejudiced outcomes or reinforcing existing disparities in patient care^21,22^. To mitigate these risks, our model incorporated diverse patient and physician attributes, and future iterations should include fairness audits, subgroup performance evaluations, and transparent reporting to ensure unbiased application in clinical settings.

A strength in our study was the direct pairing of patient and physician responses, allowing for a more accurate assessment of perception gaps in real-world clinical interactions compared with studies using unmatched samples in cross-sectional surveys. The use of multiple validated instruments and a novel questionnaire further strengthen our findings. However, the study was limited to a single center, and the sample size for epilepsy was relatively small, which may affect generalizability. A further limitation is the use of PRO data in machine learning models, as PROs are inherently subjective and may vary across individuals and contexts, potentially introducing variability that could affect model generalizability. Additionally, the PROs and the original questionnaire were not validated for physician use, with the exception of the SDM-Q-Doc, which may influence the interpretation of perception gaps.

Identifying patient characteristics associated with perception gaps may help physicians anticipate where these gaps are likely to occur in clinical practice, thereby supporting more aligned treatment planning and goal setting. The application of machine learning to predict perception gaps could offer a promising avenue for clinical decision support. However, implementation of the prediction model in the clinic will require further data accumulation (i.e. larger and diverse datasets) and external validation for generalization to different clinical settings, as well as the development of an intuitive interface for practicality and user-friendliness. Further research should explore whether perception gaps are associated with clinical outcomes such as adherence, satisfaction, or disease progression. Longitudinal studies could assess how these gaps evolve over time and whether interventions, such as communication training or decision aids, can reduce them.

In conclusion, the GAP-AI study highlights the existence and complexity of perception gaps between patients with neurological diseases and their treating physicians. These gaps are influenced by multiple factors (e.g. caregiver status, disease severity, type of diagnosis, physician’s experience level, and consultation time) and can be predicted using machine learning. The application of our prediction model showcases the potential for AI to enhance clinical decision-making processes. Recognizing and addressing perception gaps may enhance patient-centered care, potentially leading to improved outcomes in managing neurological diseases. Our data and insights may open new possibilities in patient care.

## Methods

### Study design

This was a prospective observational study involving patients with neurological diseases and their treating physicians at the Juntendo University Hospital (Tokyo, Japan). The study design was based on previous studies that evaluated perceptions of patient health status between patients and physicians^10,13,14,23^. Patients and their physicians answered questionnaires on PROs and OROs at two regular outpatient check-up visits. Patients completed the questionnaires during each visit, whereas physicians completed the questionnaires immediately after each visit and answered only once for common items between questionnaires that were not patient-dependent. Differences between questionnaire responses for each patient and their treating physician were analyzed as ‘gaps’. Although the validity of physicians providing responses to a PRO and patients providing responses to an ORO has not been evaluated, this approach has been undertaken in other studies focusing on perception gaps between patients and physicians^8,9,14^, and was considered necessary and appropriate for achieving the objectives of this study.

The study protocol and all amendments were approved by the Juntendo University Hospital ethics committee (approval number E22-0162). The study was conducted according to the ethical principles of the Declaration of Helsinki. Informed consent was obtained from each patient at visit 1 before participating in the study. This study is registered on the Japan Registry of Clinical Trials: jRCT1030220258 (https://jrct.mhlw.go.jp/en-latest-detail/jRCT1030220258).

### Study population

Patients with Parkinson’s disease, multiple sclerosis, or epilepsy visiting the Department of Neurology at Juntendo University Hospital for at least 3 months and who visited at least once in the past 3 months were eligible for inclusion. Patients were excluded if they were under 18 years of age at the time of consent, unable to answer questionnaires (e.g., owing to cognitive dysfunction), or deemed unsuitable for inclusion by the study investigator.

### Procedures

Patients and their treating physicians were given validated questionnaires to capture their perceptions of patient satisfaction (PSQ-18, SDM-Q-9/SDM-Q-Doc)^24^, ADLs (Barthel Index) and patient QoL (36-item Short Form [SF-36]; Supplementary Table 6). Additionally, disease-specific scales were used for items that could not be generalized across different neurological conditions. These included the Parkinson’s Disease Questionnaire-39 (PDQ-39)^25^, Movement Disorder Society Unified Parkinson’s Disease Rating Scale (MDS-UPDRS)^26^, Hoehn and Yahr scale^27^, Multiple Sclerosis Quality of Life-54 (MSQOL-54)^28^, EDSS^29^, and Patient-weighted Quality of Life in Epilepsy (QOLIE-31-P)^30^. Physician information (e.g., age, sex, specialist qualification, years of experience, number of patients treated) was collected separately. An original questionnaire was also distributed to assess items for which no validated assessment scale exists.

Responses to questionnaires were obtained at two outpatient visits (visit 1 and visit 2), which followed each patient’s regular appointments. Patients provided informed consent and were enrolled in the study at visit 1. Visit 2 was defined as the next scheduled appointment visit. No evaluations were performed between visits. Patients and physicians each inputted their responses onto an electronic device. Data were managed on a cloud-based system and collected through an Electronic Data Capture (EDC) platform (hashPeak, Tokyo, Japan), which employs blockchain technology to ensure data integrity and security. Data entries were timestamped, encrypted, and stored in a decentralized ledger, allowing for secure tracking and auditing throughout the study.

### Outcomes

The primary outcome was the difference between scores of pairwise items in the questionnaires (PSQ-18, SDM-Q-9/SDM-Q-Doc, Barthel Index, and the original questionnaire) answered by both patients and physicians (i.e. perception gap). Patients and physicians were also asked to rank the SF-36 subdomain that they prioritized as the most important in terms of their or their patient’s QoL, respectively.

The PSQ-18 is a tool used to assess patient satisfaction with healthcare services. It measures general satisfaction, interpersonal manner, communication effectiveness, financial consideration, duration of interaction, and ease of access^31^. The SDM-Q-9 and SDM-Q-Doc are questionnaires that measure the extent to which patients are involved in the process of decision-making and the extent to which physicians involve patients in the decision-making process, respectively^32^. The Barthel Index is an ordinal scale that measures a person’s ability to complete ADLs, including feeding, bathing, grooming, dressing, bowel control, bladder control, toileting, chair transfer, ambulation, and stair climbing^33^. The SF-36 is a commonly used questionnaire that measures overall health and well-being. It assesses eight different subdomains: physical functioning, role physical, bodily pain, general health, vitality, social functioning, role emotional, and mental health^34^. The original questionnaire developed for the current study was used to assess items for which no validated assessment scales exist. This included items about patient characteristics, disease, or treatment questions, the relationship with the physician, the physician’s clinical policy, and feelings about the medical practice.

Secondary outcomes included factors influencing perception gaps in patient satisfaction, SDM, assessment of ADLs, pairwise items assessed in the original questionnaire, and QoL that were identified in the primary outcome.

Data on potential factors influencing perception gaps were collected using disease-specific tools, including the PDQ-39 and MDS-UPDRS for Parkinson’s disease; MSQOL-54 for multiple sclerosis; QOLIE-31-P for epilepsy; the full SF-36 survey^34^, original questionnaire, and medical record data for all patients (regardless of disease); and physician information.

An exploratory outcome was the development and evaluation of a predictive model for patient–physician gap recognition using AI machine learning.

### Statistical analysis

A total sample size of 200 patients (Parkinson’s disease, 140; multiple sclerosis, 40; and epilepsy, 20), based on the historical number of outpatients at the Juntendo University Hospital, as well as 13 physicians were planned for recruitment. All enrolled participants, excluding those who withdrew consent and refused use of their data or had no data recorded after enrollment, were included for study analyses. Questionnaire items that were not answered or could not be obtained were treated as missing values. Missing values were neither assigned nor imputed. Patient and physician responses were analyzed as individual and total scores. For the primary outcome, the ‘sum of the relative difference in patient and physician responses (Yr)’ to each questionnaire was calculated using the following formula.

$$Yr=\left(Xp1-Xi1\right)+(Xp2-Xi2)+\dots +\left(Xpk-Xik\right)+\dots +(Xpn-Xin)$$

k: Question k, n: Number of questions in the questionnaire, Xpk: The kth question score of the questionnaire answered by the patient (according to the scoring of the questionnaire), Xik: Score of question k of the questionnaire answered by the physician (according to the scoring of the questionnaire).

Because subtracting a patient response from a physician response can result in a negative or positive value, there is potential for scores of individual questions to offset each other when summing across the entire questionnaire for obtaining the ‘relative difference’. For this reason, we also calculated the ‘sum of the absolute difference between patient and physician responses (Ya)’ to each questionnaire using the following formula, where paratheses of the Yr formula are replaced with absolute value signs to obtain a positive value:

$$Ya=\left|Xp1-Xi1\right|+\left|Xp2-Xi2\right|+\dots +\left|Xpk-Xik\right|+\dots +|Xpn-Xin|$$

In addition to the primary analysis, the total score of patients (Yp) and the total score of physicians (Yi) were calculated. Both total scores were defined as the sum of the scores of individual questions of each questionnaire. Differences between the total scores of patients and physicians were compared using the Mann–Whitney U test. The degree of correlation between patient and physician responses for each questionnaire was calculated using the kappa coefficient.

The distribution of the perception gap was classified into ‘concordant’ and ‘discordant’ groups, based on the median value of the Ya. Differences smaller than or equal to the median were included in the concordant group and differences larger than the median were included in the discordant group. A cross-tabulation analysis of patient and physician background factors was performed between the concordant and discordant groups to determine their potential influence on the perception gap using Fisher’s exact test.

Correlations of differences between questionnaires (except for SF-36 subdomains) in terms of the Ya were evaluated using Spearman’s correlation coefficients. For the correlation of the SF-36 subdomain with other questionnaires, summary statistics for concordance/discordance of the SF-36 subdomain with each questionnaire in terms of the Ya were calculated and compared using the Mann–Whitney U test.

A univariate analysis was conducted to assess the association between patient characteristics and physician attributes that could potentially influence the perception gap using the Spearman’s correlation coefficient. A subsequent multiple regression analysis was performed using disease type as the dependent variable and significant patient characteristics/physician attributes (*P* < 0.05; as determined from the univariate analysis) as independent variables.

SPSS Statistics 26 (IBM Corporation, Armonk, NY, USA) was used for statistical analyses.

### Model development

The patient and physician data set was used to develop and evaluate predictive models for recognizing perception gaps between patients and physicians. A supervised machine learning model was developed and trained on primary outcome data and secondary outcome response data. The perception gap was treated as a binary variable (concordant and discordant groups). To ensure robust cross-testing and efficacy in predicting perception gaps, we employed multiple machine learning algorithms, which included k-nearest neighbors, random forest, ensemble methods, neural networks, logistic regression, boosting, linear support vector machines, decision trees, and naïve bayes.

The target of the analysis was the difference between the patient and physician scores for the PSQ-18, SDM-9-Q/SDM-Q-Doc, Barthel Index, the original questionnaire (pairwise items evaluated by patients and physicians), and the responses to the SF-36 subdomain. The features of the learning model were patient and physician total scores of PSQ-18, SDM-9-Q/SDM-Q-Doc, Barthel Index, the original questionnaire, and the SF-36 subdomain; physician attribute information; patient-specific questionnaires (excluding patient–physician pairwise assessments and disease-specific assessments); and relevant medical record data. To enhance model inference accuracy, we performed feature selection using SHAP values to identify and retain the top 15 most influential features. This helped to reduce dimensionality while preserving the most predictive variables. To improve model training stability and generalization, the dataset was duplicated to increase the number of epochs. This approach aimed to mitigate overfitting and enhance model robustness in a high-dimensional context with small sample sizes.

### Model validation

To evaluate model accuracy, fivefold cross-validation was used, with each fold serving once as a validation set while being trained on the remaining folds. Additionally, 20% of the data were withheld as a separate test set to further assess model generalization. Model performance was measured using log loss, which evaluates the degree to which predicted probabilities diverge from actual class labels, and AUC-ROC, indicative of the model’s ability to distinguish between classes^35^. To ensure the best balance between true and false positives and negatives, the Matthew’s correlation coefficient (MCC) was considered for selecting the classification threshold using the following formula^36^.

$$MCC=\left(\left(TP\times TN-FP\times FN\right)\right)\div \surd (\left(TP+FP\right)\left(TP+FN\right)\left(TN+FN\right))$$

TP: True Positive, TN: True Negative, FP: False Positive, FN: False Negative.

Initial hyperparameter values were adopted from default settings provided by the respective machine learning libraries. Subsequent tuning was conducted on the model with the lowest log loss to refine performance, utilizing a grid search approach over a defined parameter space. This process was iteratively performed with varying parameters to identify the model configuration with the highest predictive accuracy. The final model development and analysis were conducted using Python 3.8.17 (Python Software Foundation).

## Supplementary Information

Supplementary Information.

## Data Availability

The datasets used and/or analyzed during the current study are available from the corresponding author (g_oyama@juntendo.ac.jp) on reasonable request.
