# Supplementary material for: Identification of perception gaps between physicians and patients with neurological diseases and the prediction of these gaps using machine learning
Source: Sci Rep. 2026 Feb 9;16:5394. doi: 10.1038/s41598-025-33500-x (PMC12886985; doi:10.1038/s41598-025-33500-x)

Supplementary information

Identification of perception gaps between physicians and patients with neurological diseases and the prediction of these gaps using machine learning

Genko Oyama, Yuji Tomizawa, Taiji Tsunemi, Shuko Nojiri, Taku Hatano, Wataru Sako, Yasunobu Hoshino, Shin-ichi Ueno, Daiki Kamiyama, Yutaka Oji, Ayami Okuzumi, Daisuke Taniguchi, Haruna Haginiwa, Takuma Maeda, Yoshihiko Furusawa, Miwa Izutsu, Nobutaka Hattori

Supplementary Table 1. Patient demographics and baseline characteristics by disease.

	**Parkinson's disease n = 137**		**Multiple sclerosis n = 40**		**Epilepsy n = 20**
**Age, mean (SD), years**	66.4 (9.6)	**Age, mean (SD), years**	45.6 (8.8)	**Age, mean (SD), years**	26.6 (8.9)
**Female, n (%)**	75 (54.7)	**Female, n (%)**	31 (77.5)	**Female, n (%)**	13 (65.0)
**Disease duration, mean (SD), years**	7.4 (6.1)	**Disease duration, mean (SD), years**	8.8 (6.4)	**Disease duration, mean (SD), years**	4.3 (5.3)
**Disease duration, n (%)**		**Disease duration, n (%)**		**Disease duration, n (%)**	
< 5 years	50 (38.5)	< 5 years	8 (21.1)	< 5 years	13 (68.4)
5–10 years	37 (28.5)	5–10 years	15 (39.5)	5–10 years	3 (15.8)
≥ 10 years	43 (33.1)	≥ 10 years	15 (39.5)	≥ 10 years	3 (15.8)
**Hoehn and Yahr stage, n (%)**		**MSQOL-54, mean (SD)**		**Seizure frequency in last 3 months, n (%)**	
0	2 (1.5)	Physical health	69.0 (14.8)	0	15 (75.0)
I	15 (10.9)	Mental health	67.1 (15.4)	1	5 (25.0)
II	96 (70.1)	**EDSS, n (%)**		≥ 2	0
III	19 (13.9)	0	12 (30.0)	**QOLIE31-P, mean (SD)**	75.1 (16.3)
IV	4 (2.9)	0.5	0	**Adverse events from antiepileptic drugs, n (%)**	
V	1 (0.7)	1.0	6 (15.0)	Dizziness	2 (10.5)
**MDS-UPDRS total score, mean (SD)**	41.3 (19.8)	1.5	5 (12.5)	Somnolence	10 (52.6)
Part I	9.7 (6.0)	2.0	9 (22.5)	Headache	5 (26.3)
Part II	10.7 (8.2)	2.5	2 (5.0)	Gastrointestinal symptoms	3 (15.8)

Part III	18.4 (10.5)	3.0	2 (5.0)	Anorexia	0
Part IV	2.7 (3.1)	3.5	1 (2.5)	Overeating	2 (10.5)
**PDQ-39 summary index, mean (SD)**	18.8 (15.0)	4.0	0	Hair loss	1 (5.3)
		4.5	0	Dermatological symptoms	0
		5.0	0	Fatigue	4 (21.1)
		5.5	0	Fever	0
		6.0	1 (2.5)	Weight changes	1 (5.3)
		6.5	2 (5.0)	Irregular menstruation	0
				Irritability	0
				Depression	1 (5.3)

Note: Not all patient demographics or disease characteristics were calculated with the patient numbers for Parkinson's disease (n = 137), multiple sclerosis (n = 40), and epilepsy (n = 20) as the denominators.

Hoehn and Yahr stage: 0, no symptoms; I, Unilateral symptoms only; II, Bilateral symptoms but no balance disorder; III, Mild-to-moderate symptoms. Balance disorder but no physical assistance required; IV, Severe movement disorder. Able to stand or walk without assistance; V, Wheelchair or bedridden without assistance.

EDSS, Expanded Disability Status Scale; MDS-UPDRS, Movement Disorder Society Unified Parkinson's Disease Rating Scale; MSQOL-54, Multiple Sclerosis Quality of Life-54 items; PDQ-39, Parkinson's Disease Questionnaire-39 items; QOLIE-31-P, Patient-weighted Quality of Life in Epilepsy inventory; SD, standard deviation.

Supplementary Table 2. Differences in perception between patient–physician by comparing total scores and kappa coefficient.

	Mean total score			Mean kappa coefficient (SD)
	Patient (Yp)	Physician (Yi)	Mann-Whitney U test	
**PSQ-18**	64.4	61.2	< 0.001	0.030 (0.042)
**SDM-Q-9/SDM-Q-Doc**	34.1	27.0	< 0.001	0.021 (0.019)
**Barthel Index**	97.4	97.4	0.420	0.172 (0.185)
**Original questionnaire**	49.0	44.6	< 0.001	0.039 (0.040)
**SF-36 subdomain**	N/A	N/A	N/A	0.203 (0.049)[a]

[a]Asymptotic standard error.

PSQ-18; Patient Satisfaction Questionnaire-18 item; SD, standard deviation; SDM-Q-9; 9-item Shared Decision Making Questionnaire from the patient perspective; SDM-Q-Doc; Shared Decision Making Questionnaire from the physician perspective; SF-36, 36-item Short Form; Yi, total score of physicians; Yp, total score of patients.

Supplementary Table 3. Spearman's rank correlation of the sum of the absolute differences (Ya) between questionnaires.

	PSQ-18	SDM-Q-9/ SDM-Q-Doc	Barthel Index	Original questionnaire
**PSQ-18**		ρ = 0.285 (P < 0.001)	ρ = –0.072 (P = 0.347)	ρ = 0.295 (P < 0.001)
**SDM-Q-9/SDM-Q-Doc**	–		ρ = –0.049 (P = 0.522)	P = 0.400 (P < 0.001)
**Barthel Index**	–	–		ρ = 0.049 (P = 0.516)
**Original questionnaire**	–	–	–	

PSQ-18; Patient Satisfaction Questionnaire-18 item; SDM-Q-9; 9-item Shared Decision Making Questionnaire from the patient perspective; SDM-Q-Doc; Shared Decision Making Questionnaire from the physician perspective.

Supplementary Table 4. Sum of the absolute differences (Ya) of questionnaires by concordance of SF-36 subdomains.

		Ya, mean (SD)	Mann-Whitney U test
**PSQ-18**	Concordant	15.6 (5.5)	P = 0.488
	Discordant	16.5 (5.7)	
**SDM-Q-9/SDM-Q-Doc**	Concordant	12.6 (8.3)	P = 0.758
	Discordant	12.7 (7.7)	
**Barthel Index**	Concordant	2.9 (6.8)	P = 0.829
	Discordant	3.8 (10.0)	
**Original questionnaire**	Concordant	8.4 (3.5)	P = 0.701
	Discordant	8.4 (4.0)	

PSQ-18; Patient Satisfaction Questionnaire-18 item; SD, standard deviation; SDM-Q-9; 9-item Shared Decision Making Questionnaire from the patient perspective; SDM-Q-Doc; Shared Decision Making Questionnaire from the physician perspective; SF-36, 36-item Short Form.

Supplementary Table 5. Evaluation of supervised machine learning analysis: holdout log loss.

Algorithms	PSQ-18	SDM-Q-9/ SDM-Q-Doc	Barthel Index	Original questionnaire	SF-36 subdomain
**k-nearest neighbors**	0.1342	0.1709	0.0581	0.1333	0.0500
**Random forest**	0.2667	0.2304	0.1838	0.2198	0.1543
**Ensemble top3**	0.2286	0.2416	0.1595	0.2189	0.2053
**Neural networks**	0.1479	0.3844	0.2989	0.3349	0.3026
**Logistic regression**	0.4023	0.5888	0.3639	0.5833	0.4263
**AdaBoost**	0.5699	0.5946	0.4319	0.6327	0.4421
**Linear SVM**	0.3739	0.6469	0.4129	0.5946	0.4317
**Decision tree**	0.5602	1.5683	0.3863	0.6458	0.4520
**Naive bayes**	2.6356	3.6642	3.6754	2.9805	12.2924

PSQ-18; Patient Satisfaction Questionnaire-18 item; SDM-Q-9; 9-item Shared Decision Making Questionnaire from the patient perspective; SDM-Q-Doc; Shared Decision Making Questionnaire from the physician perspective; SF-36, 36-item Short Form; SVM, support vector machine.

Supplementary Table 6. Questionnaires assigned to patients and physicians.

	**Patient**	**Physician**
**Patient satisfaction**	PSQ-18 (V2)	PSQ-18 (V2)
**SDM**	SDM-Q-9 (V2)[a]	SDM-Q-Doc (V2)
**Impairment of ADLs**	Barthel Index (V2)	Barthel Index (V2)
**Quality of life**	SF-36 subdomain and the full SF-36 (V1)[b]	SF-36 subdomain (V2)
**Parkinson's disease[c]**	MDS-UPDRS (V1, V2)[b]<br>PDQ-39 (V2)	Hoehn and Yahr (V1)[d]<br>MDS-UPDRS (V1, V2)[d]
**Multiple sclerosis[c]**	MSQOL-54 (V2)	EDSS (V1)[d]<br>FS (V1)[d]
**Epilepsy[c]**	QOLIE-31-P (V1)[b]	Seizure frequency (V1)[d]<br>Symptoms of side effects of antiepileptic drugs (V1)[d]
**Original questionnaire**	Original questionnaire (V2)[e]	Original questionnaire (V2)[e]
**Other**	–	Physician attribute information<br>Medical record information

[a]The SDM-Q-9 was answered by the patient after their appointment at visit 2. It asked the patient to reflect on the consultation with their treating physician and respond to questions about the SDM process with their treating physician.
[b]If PROs could not be obtained at visit 1, information was obtained at visit 2.
[c]Physician disease-specific questionnaires included assessment on disease severity and disease-specific conditions/symptoms.
[d]Basic patient information and responses to these surveys were transcribed from electronic medical records after visit 1.

[e]Responses to one of the questions in the original questionnaire were not collected for patients with Parkinson's disease and epilepsy due to a system error. As a result, data for this question were missing for 147 patients.

ADL, activities of daily living; EDSS, Expanded Disability Status Scale; FS, Functional Systems; MDS-UPDRS, Movement Disorder Society Unified Parkinson's Disease Rating Scale; MSQOL-54, Multiple Sclerosis Quality of Life-54 items; PDQ-39, Parkinson’s Disease Questionnaire-39 items; PRO, patient-reported outcome; PSQ-18; Patient Satisfaction Questionnaire-18 item; QOLIE-31-P, Patient-weighted Quality of Life in Epilepsy inventory; SDM, shared decision-making; SDM-Q-9; 9-item Shared Decision Making Questionnaire from the patient perspective; SDM-Q-Doc; Shared Decision Making Questionnaire from the physician perspective; SF-36, 36-item Short Form; V1/V2, visit 1/visit 2.

Supplementary Figure 1. The ROC curves of target diagnoses for all tested machine learning models. PSQ-18; Patient Satisfaction Questionnaire-18 item; ROC, receiver operating characteristic; SDM-Q-9; 9-item Shared Decision Making Questionnaire from the patient perspective; SDM-Q-Doc; Shared Decision Making Questionnaire from the physician perspective; SF-36, 36-item Short Form.

PSQ-18

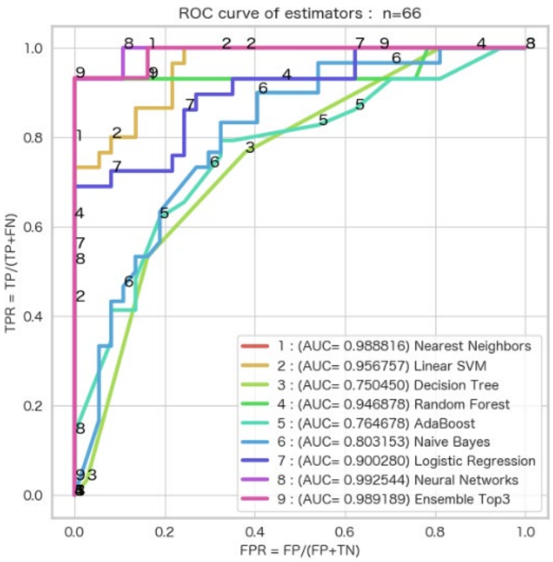

SDM-Q-9/SDM-Q-Doc

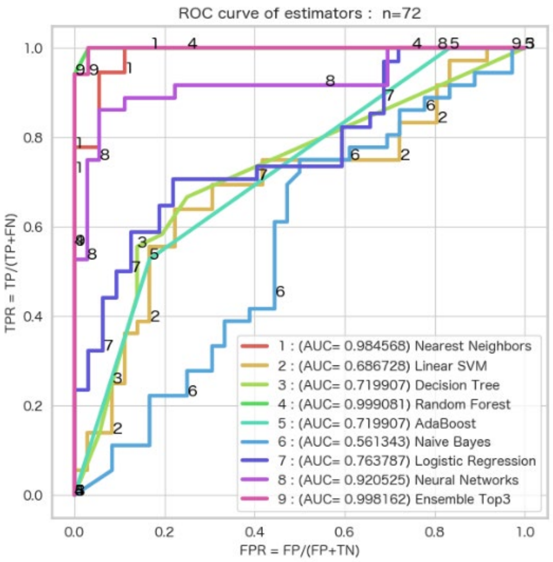

Barthel Index

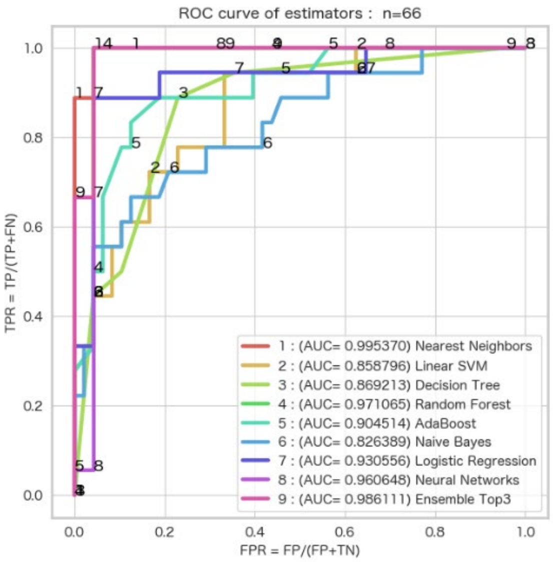

Original questionnaire

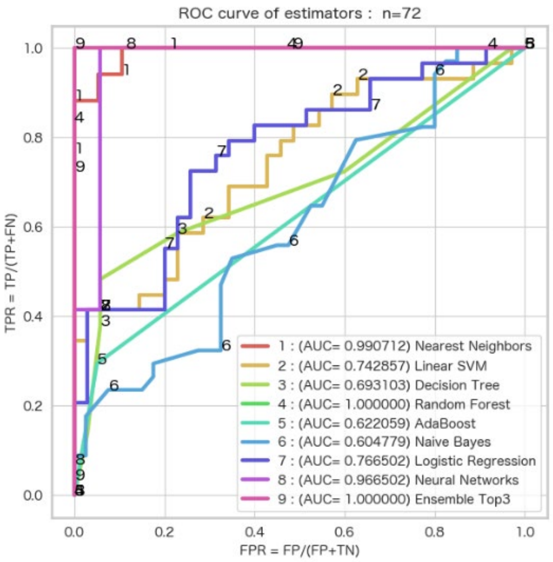

SF-36 subdomain

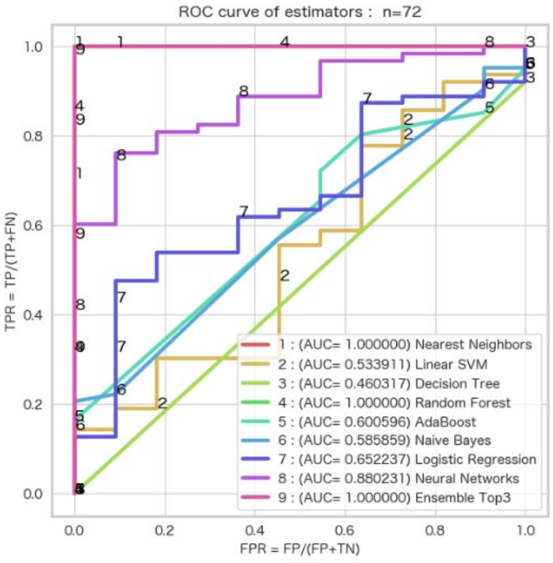

Supplement: Supplementary file 1 — Supplementary Information. [file 41598_2025_33500_MOESM1_ESM.pdf]
